# Feedback for Learning in Pharmacy Education: A Scoping Review

**DOI:** 10.3390/pharmacy9020091

**Published:** 2021-04-23

**Authors:** Nicholas R. Nelson, Rebecca B. Carlson, Amanda H. Corbett, Dennis M. Williams, Denise H. Rhoney

**Affiliations:** 1 Division of Practice Advancement and Clinical Education, UNC Eshelman School of Pharmacy, Chapel Hill, NC 27599-7475, USA; nnelson2@email.unc.edu; 2 Health Sciences Library, University of North Carolina at Chapel Hill, Chapel Hill, NC 27599-7585, USA; rcarlson@unc.edu; 3 Division of Pharmacotherapy and Experiential Therapeutics, UNC Eshelman School of Pharmacy, Chapel Hill, NC 27599-7569, USA; ahcorbet@email.unc.edu (A.H.C.); dwilliams@unc.edu (D.M.W.)

**Keywords:** feedback, pharmacy education, scoping review

## Abstract

Feedback is an effective pedagogy aimed to create cognitive dissonance and reinforce learning as a key component of clinical training programs. Pharmacy learners receive constant feedback. However, there is limited understanding of how feedback is utilized in pharmacy education. This scoping review sought to summarize the breadth and depth of the use of feedback within pharmacy education and identify areas for future research. PubMed, Embase, Scopus, and Web of Science were searched for English articles since January 2000 to identify studies related to feedback in pharmacy education. Sixty-four articles were included for analysis, stratified by moderate and major theory talk, where moderate theory talk explicitly included feedback into study design and major theory talk included feedback into both study design and analysis. Feedback was provided in Bachelor (14%), Master (15.6%), Doctor of Pharmacy (67.2%) and post-graduate programs (4.7%) on a variety of curricular objectives including communication and patient work up in didactic, objective structured clinical examination (OSCE), and experiential settings, and career/interview preparation in the co-curriculum. Feedback comments were mostly written in didactic courses, and both written and verbal in OSCE, experiential, and co-curricular settings. The pharmacy education feedback literature lacks depth beyond student perceptions, especially with respect to assessing the effectiveness and quality of feedback for learning. While feedback has been utilized throughout pharmacy education across myriad outcomes, several areas for inquiry exist which can inform the design of faculty and preceptor development programs, ensuring provision of effective, quality feedback to pharmacy learners.

## 1. Introduction

Feedback has been identified as a critical component of clinical training programs [1]. It has been described as “the heart of medical education” [2] and “the cornerstone of effective clinical training” [3]. The Accreditation Council for Pharmacy Education highlights the importance of the provision of feedback in both didactic and experiential curricula, requiring formative performance feedback to students in both settings [4]. Additionally, the American Society of Health-System Pharmacists describes feedback as “essential for residents’ skill development” and requires verbal formative feedback by Standard 3.4 in their guidance document for postgraduate year-one pharmacy residency program accreditation standards [5].

Feedback has been proposed to play a critical role at the center of adult learning theory and is among the most influential moderator of learning [6,7]. The main roles of feedback are to identify and minimize student dissonance and reinforce correct learning for consolidation into existing knowledge and skill development. Feedback can have many effects in addition to confirming the accuracy of a student including increasing student effort and motivation to engage in learning, exposing different methods of understanding to the student, and conveying progress toward a student’s goal. While feedback has been shown to be one of the most powerful tools in the teaching arsenal, a meta-analysis reported that one-third of feedback had a negative impact on achievement, possibly due to person-focused feedback as opposed to process or task-oriented feedback [8]. This highlights the critical, yet variable impact feedback can have on learning [6].

Pharmacy learners are constantly being provided feedback from various sources including faculty, standardized patients, preceptors, and peers throughout their education. This feedback is also delivered to students in many forms including written, verbal, and automatic response devices. Most of the discussion involving feedback within the health professions education literature, however, is described in other disciplines, primarily medicine [9]. Although pharmacy learners were incorporated in a prior scoping review of feedback in medical education, the number of publications regarding feedback to pharmacy learners has increased and it has not been evaluated broadly within the pharmacy education literature [9]. The purpose of this scoping review was to identify the breadth and depth of evidence regarding the use of feedback for learning in pharmacy education and training and identify potential gaps which could lead to future research.

## 2. Methods

This scoping review sought to determine what has been published regarding the provision of feedback within pharmacy education to improve student knowledge/competency. For this review, feedback was defined as information written or verbally communicated to a learner from a teacher/preceptor that is intended to modify the learner’s thinking or behavior for the purpose of improving learning. The five-phase procedure for scoping reviews was utilized: (1) identify the research question; (2) identify relevant studies; (3) select relevant articles; (4) chart the data; and (5) collate, summarize, and report the results [10]. A research team of investigators with experience in pharmacy education, literature searching, and scoping reviews was established for this review.

This scoping review focused on addressing the question: “What is known about the use of feedback provided to pharmacy students and trainees on learning?” The goal was to identify key concepts, gaps in the literature, and sources of evidence to inform practice and potential avenues of research in pharmacy education. This scoping review did not seek to evaluate the quality of the present literature or develop recommendations on best practices regarding the provision of feedback.

The initial search and an updated search were conducted on 6 September 2019 and 19 March 2021, respectively, using PubMed, Embase, Scopus, and Web of Science for literature published from 1 January 2000 forward. The search query consisted of terms related to education or learners, pharmacy, and feedback using medical subject headings or Emtree subject headings where available and keywords when applicable (Supplementary Table S1). All citations identified by the search strategy were imported into Covidence (Veritas Health Innovation, Melbourne, Australia) after removing duplicates. Citations then underwent a two-stage screening process consisting of title/abstract review using an abstract screening form and full-text review by two authors for inclusion. Letters to the editor, editorials, commentaries, conference abstracts, and non-English language articles were excluded. Peer-only feedback was also excluded as peer tutoring and peer influences are considered to be distinct influence with different effect sizes [11]. Articles that did not have an abstract were included for full-text review if the title indicated inclusion. All conflicts were resolved by an independent third author. Included citations then underwent a similar process for theory talk analysis adapted from Kumasi et al. and Lyons et al. to qualitatively analyze the extent to which authors utilized feedback (Table 1) [12,13]. To specifically focus on the role of feedback on learning, only moderate and major theory talk citations were included. Each author participated in data extraction using a spreadsheet created to chart data in Microsoft Excel Version 16.41 (Microsoft, Redmond, WA, USA), which included the following categories: author; year of publication; sample size; objective, intervention, and results of study; level of learner; method, setting, focus area, and assessment of feedback; and “other” given the heterogeneity of article types included in the analysis. Once data were charted in the spreadsheet, the authors met to discuss key themes that were identified.

## 3. Results

**Figure 1:**
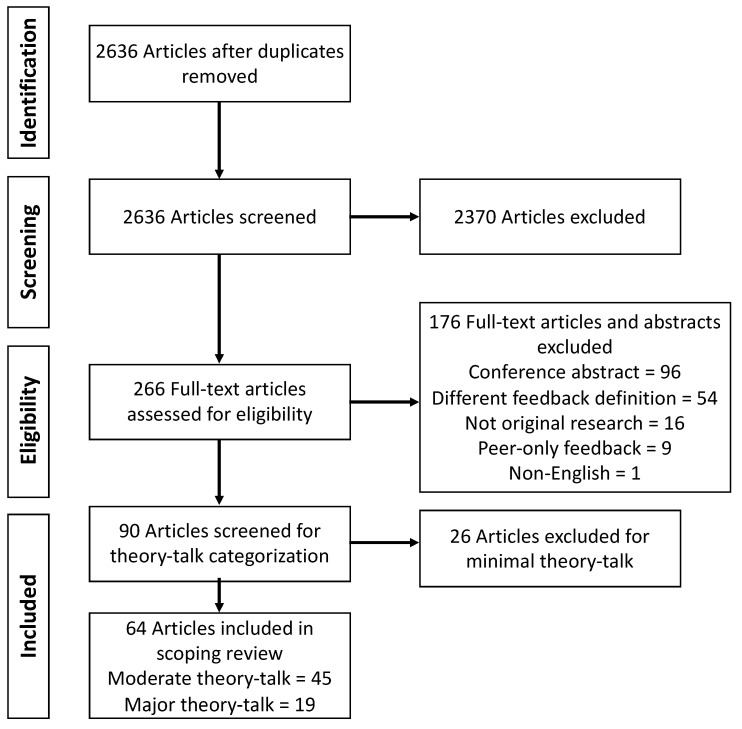
Preferred Reporting Items for Systematic Reviews and Meta-Analyses (PRISMA) flow diagram for scoping review search strategy.

Figure 1 shows the PRISMA flow chart for article inclusion. Overall, 64 citations were included for analysis in this review from around the world encompassing Bachelor (BPharm), Master (MPharm), and Doctor of Pharmacy (PharmD) programs spanning four curricular settings: didactic, objective structured clinical examination (OSCE), experiential, and co-curriculum (Table 2) (see Supplementary Tables S2–S5 for full data extraction). Feedback was provided for a variety of topics including communication, patient work up, and career/interview preparation through written, verbal, or multimodal (written and verbal) means (Table 3).

### 3.1. Didactic Studies

Over half of the included studies focused on feedback given in didactic settings. These 36 studies include 24 moderate theory talk while 12 were categorized as major theory talk. They span the didactic curricular setting, including students from the first to final years of Bachelor (BPharm), Master (MPharm), and Doctor of Pharmacy (PharmD) programs, and range in study size from 18 to 621 participants. Most of the studies in this setting provided written feedback, while smaller percentages provided verbal or multimodal feedback (Table 3).

The moderate theory talk articles examined new didactic courses and course interventions, and included feedback on student performance within their design, but did not assess the given feedback for quality or impact. Overall, as a group, these papers did not focus on feedback as a key study objective or outcome; only four studies included feedback in their study objectives, but no aspect of the feedback provided was included for analysis [14,15,16,17]. These four papers tested the use of feedback in three different areas: improving CPR skills development [14]; subjective, objective, assessment, and plan (SOAP) note writing [15]; and student performance on verbal competency and patient counseling and interviewing assessments [16,17]. The other studies’ feedback focus areas were patient communication including counseling and other simulated patient interactions [18,19,20,21,22]; patient work up including SOAP notes [18,23,24,25,26,27,28] or oral case presentations and care plans [26,29,30,31]; and other topics such as evidence appraisal and research skills [32,33]; self-assessments [34]; disease information [35]; pharmacy calculations [36]; and pharmacology experiments [37].

Eleven of the 12 major theory talk articles assessed the impact of feedback on student learning. Seven articles evaluated the impact of a singular method of delivering feedback [audio-verbal [38,39], objective rubric [40], online (*n* = 90 s and third-year PharmD (P2-P3) and 410 first-year PharmD (P1) students over 5 years) [41,42], or written (*n* = 133 fifth-year BPharm students and 150 MPharm) [43,44] on learning whereas four articles compared multiple methods of delivery including online vs. handwritten (*n* = 169 first-year MPharm and 201 P3) [45,46], verbal vs. written (350 P2-P3) [47], and audio-verbal vs. written (*n* = 75 P1) [48]. Only one major theory talk article evaluated the quality of feedback provided, comparing peer to faculty feedback (*n* = 182 fourth-year BPharm) [49].

Audio-verbal feedback and the use of an objective rubric resulted in positive outcomes with respect to compression depth in 120 “novice” learners, and all compression and ventilation outcomes for 104 “novice” learners during CPR training [38,39] and SOAP note grading and standardized patient checklist over successive cases for 126 P3 students [40], respectively. Studies evaluating online feedback yielded varying results on feedback’s impact on learning; one study found it promoted improvements on future SOAP notes [41], while another found mixed results as significant grade improvements occurred between two cohorts, but four cohorts had no difference [42]. One study found students engaging with written feedback as part of problem-based learning significantly increased laboratory practical grades [43]. Similarly, another study saw an increase in student pre- and post-course patient work-up scores [44]. Some evidence suggests that written online feedback was more timely than written paper feedback [46] while audio-verbal feedback took 1.5 times longer than written [48]. However, in terms of amount of feedback, praise and error identification, and personability, more feedback was provided through audio-verbal and online than paper [45,48]. Alternatively, the role of multimodal feedback in improving student problem-solving skills compared to a singular delivery method or no feedback at all was demonstrated [47]. Finally, peer assessment resulted in higher grades than experts. However, there was no difference in the quality of feedback provided [49].

### 3.2. Objective Structured Clinical Examination/Simulation Studies

There were seven articles (five moderate theory talk and two major theory talk) related to OSCE or simulation activities and all but one included learners in the later years of the pharmacy curriculum. One moderate theory talk article described the development and design of a formative and summative OSCE program across the entire curriculum [50]. All five moderate theory talk studies in a simulation or OSCE training environment incorporated immediate verbal [51,52,53] written [50], or multimodal [54] feedback into their methodology.

One major theory talk article sought to compare immediate versus delayed feedback after a patient counseling simulation for 153 P3 students. While the trainees preferred the immediate feedback, overall grades did not differ between immediate and delayed feedback [55]. Learner satisfaction and feedback preferences of 20 final year MPharm students was compared with three scenario simulation modalities [49]. The paper-based simulation had feedback provided immediately in the form of model answers to written questions and a small group discussion. The computer-based simulation incorporated feedback immediately after completing the game as a detailed scorecard. For the simulated patient, a video recording of the role-play was provided to the students along with their score and feedback using a guide the day after the experience [56].

### 3.3. Experiential Studies

Twelve of the included studies focused on feedback given in experiential settings including seven moderate theory talk [57,58,59,60,61,62,63] and five major theory talk [64,65,66,67,68]. All articles included learners in the final year of their pharmacy program ranging from 13 to 162 participants. Two articles compared early learners or post-graduate trainees to learners in the final year of the program [58,60]. Most of the studies provided multimodal feedback while the remainder provided written feedback only (Table 3).

The moderate theory talk articles primarily examined feedback on learner communication through patient medication history and counseling [58,62] or motivational interviewing [57], patient work up via simulated patient case scenarios [60,61], or student knowledge of pharmaceutical calculations [59]. One article focused feedback to residents on their provision of feedback to students [63]. The primary format for feedback in these studies was multimodal [57,58,59,60] while other studies provided only written [61] or verbal feedback [62,63]; however, none of the studies examined the direct impact of feedback on learning.

In contrast, the four major theory talk articles directly assessed learner feedback using multimodal [66] or written only approaches [64,65,67]. These studies examined feedback as it relates to students’ patient counselling skills, problem solving, clinical care (assessment/plan), evidenced-based medicine application, professionalism, communication, effective student self-reflections, and SOAP note writing. In all major theory talk studies, student performance (knowledge or abilities) was improved as a result of the feedback [64,65,66,67]. Feedback increased student scores across three SOAP notes after written feedback was provided to 54 fourth-year PharmD (P4) students. In addition, there was positive correlation between SOAP note performance and advanced pharmacy practice experience (APPE) grade [64]. The impact of feedback on student achievement of curricular outcomes (patient-centered assessment and plan, evidence-based medicine application, professionalism, and communication) was evaluated in another study of 149 students by utilizing faculty feedback to students. Ninety-seven percent of students in their APPE year demonstrated achievement of these ability-based outcomes [65]. Another study showed communication and counseling skills of 45 fourth-year BPharm students were significantly improved over three sessions as a result of both verbal and video recorded feedback compared to no feedback [66]. Another evaluation demonstrated that, among 34 students, providing feedback on reflective responses during an ambulatory care APPE led to more “reflective” responses (intervention) as compared to less “reflective” responses when no feedback was given (control) [67]. A final study assessed SOAP note performance finding that 128 P4 students performed better on a second SOAP note after written feedback in all sections. However, semester of APPE had no effect on performance [68].

### 3.4. Co-Curriculum Studies

Finally, nine studies included feedback within the co-curricular space, all of which were moderate theory talk [69,70,71,72,73,74,75,76,77]. Five studies provided only written feedback [69,73,75,76,77] while three studies provided multimodal feedback [70,71,74], and one study provided only verbal feedback [72]. The focus of most publications was related to preparing final year students in obtaining a residency or employment through mock interview practice, curriculum vitae development, and related activities [69,71,72,73,74,78] while two focused on student reflections [75,76], and one incorporated feedback into a student portfolio activities [77]. No study evaluated the quality or impact of feedback provided, only reporting student perceptions on the feedback they received. Although three studies included more than 100 participants [74,75,77], samples sizes in individual studies were generally small ranging from 9–39 participants [69,70,71,72,73,76].

### 3.5. Identified Gaps

Several gaps have been revealed from this scoping review. First, documentation and evaluation of the quality of feedback is largely absent in the pharmacy education literature as only 1 of 64 included studies assessed the quality of feedback provided. However, this study evaluated feedback quality to compare faculty comments to peer comments, finding no difference in quality of feedback between faculty and peer [49]. Second, as mentioned above, the impact and effectiveness of the various feedback interventions on student learning and performance is largely unknown as authors tend to focus on student perception data. In addition, few studies have assessed the use of feedback in the post-graduate setting. No studies included trainees in fellowship programs and only four of 64 (6.25%) included learners in clinical training programs. There are also areas to explore feedback within interprofessional education as only one major theory talk article included multidisciplinary learners and two moderate theory talk articles evaluated pharmacy and either neuroscience or advanced practice nursing students. No articles assessed pharmacy and other health profession learners. Finally, given that only professional development and student reflection skills were studied in the co-curricular setting, and the impact of feedback was not included, there are numerous opportunities for in depth research as well as a broader scope of research in this area. The use and role of feedback in both patient care and non-patient care activities including public health outreach and education events, interprofessional education, leadership development, and cultural competency is still largely unknown.

## 4. Discussion

This scoping review summarizes what has been published about feedback as it relates to information written or verbally communicated to a learner from a teacher/preceptor that is intended to modify the learner’s thinking or behavior for the purpose of improving learning in pharmacy education. Importantly, from the initial search it is clear that “feedback” is a very broadly used term as only 10% of identified articles met criteria for full-text review. Furthermore, of those that underwent full-text review, 20.3% used “feedback” to describe an intervention or program different than how this review defined “feedback,” warranting exclusion from analysis.

The breadth of feedback utilized throughout pharmacy education and training around the world is wide reaching. The 64 included articles span the entirety of pharmacy education from first programmatic year of Bachelor, Masters, and Doctor of Pharmacy programs to postgraduate year two residency programs. The majority of these studies came from the United States and focused on students in the didactic years/courses of their curriculum while 12 articles studied feedback in the experiential curriculum including three articles that included postgraduate training. There are also several ways that feedback was provided to learners including written and verbal, or a combination of both. The areas in which feedback was focused also encompasses many key elements of pharmacy training. Articles from didactic, OSCE, and experiential curricular settings focused on similar topics including communication and patient work up. The co-curricular setting, however, focused exclusively on professional development in the form of career/interview preparation or reflection, primarily for final year students seeking residency training. Didactic articles primarily included written feedback whereas multimodal feedback was more common in OSCE, experiential, and co-curricular settings. This is likely due to the fact that didactic courses typically have larger student to feedback provider ratio compared to the other settings making verbal feedback less feasible compared to OSCE or experiential settings. It is worth noting, however, the relatively few articles of feedback during OSCEs given its effectiveness in this environment [79].

The included articles in this review demonstrate the relative paucity of utilization and impact of feedback within the pharmacy education literature. Much of what is known about feedback and how to provide feedback in pharmacy, dental, and medical education is based on little to no high-quality evidence, thus evidence-based recommendations are lacking [9,80]. A recent systematic review of feedback within nursing education, however, found quality feedback as described by students to be timely, incorporate positive and constructive comments, be directly related to the content, linguistically clear, and feed forward or justify a grade [81]. Importantly, studies which included an assessment of student performance all found positive impact from their educational interventions although evidence suggests that up to one-third of feedback can have a negative effect [8]. This may be due to feedback rarely being the sole intervention or publication bias. Although this review did not intend to assess the quality of included articles, the small number of articles which not only described how feedback was incorporated in the educational intervention, but also assessed its impact on student learning is astonishing. Only 19 of the 90 (21.1%) articles screened for theory talk analysis met major theory talk criteria where feedback was not only included in the educational design, but its use was also analyzed as part of the study. Therefore, feedback appears to more commonly be an aspect of an educational intervention but has rarely been assessed for impact, effect size, or quality. In addition to the lack of feedback assessment, most of the analyzed articles in the experiential and co-curricular settings had relatively small sample sizes, especially compared to feedback in the didactic or OSCE settings. This may be due to the fact that not every learner will take the same experiential rotation and many of the co-curricular studies were primarily voluntary and consisted of students seeking residency training, possibly limiting generalizability pharmacy learners at large. Finally, while articles seeking only to evaluate student perception of feedback were excluded, contrary to their main objective, results often focused on student perception data as opposed to the impact, effectiveness, or quality of the feedback intervention in concordance with their stated objectives, further limiting the depth of feedback literature.

There are some notable limitations to this review. First, the used definition of “feedback” may have resulted in an overly narrow group of included articles which was further limited through applying theory talk analysis due to potential author bias. Given the common use, range of definitions of feedback, and large number of articles initially identified in the initial database search, however, this review was specifically designed to describe feedback from an educator to a learner. In addition, all articles underwent independent review by two authors with a third author resolving any conflicts to maintain objectivity. However, data extraction was not confirmed by a second author, so varying results are possible if different or additional investigators were involved in thematic analysis. This subgroup of articles was also limited to only articles written in English. Only one article was excluded for this reason, however, which increases confidence in the search strategy for a complete evaluation of the breadth and depth of feedback related literature.

## 5. Conclusions

Feedback is an important educational tool to be leveraged within clinical training programs. This review demonstrated the wide breadth of feedback literature within all levels of pharmacy education around the world. Feedback can clearly be incorporated into multiple curricular settings as part of course designs to focus on the improvement of a myriad of knowledge and skills. An important finding from this review, however, is the lack of depth to which feedback has been studied within pharmacy education. Although feedback is commonly incorporated in educational interventions, it is rarely a primary focus of studies and its impact is not typically assessed. Several gaps and opportunities for further inquiry have been identified through this scoping review. The effectiveness and quality of feedback in both patient and non-patient care activities in didactic, experiential, and co-curricular learning beyond student perceptions are two areas ripe for research in addition to post-graduate and interprofessional education. Answers to these gaps could help inform the design of training programs for faculty to deliver quality feedback to students, increasing its positive effects.

## Figures and Tables

**Table 1:** Analytic theory talk continuum categories.

Theory Talk Classification	Analytical Category	Definition of Category
Minor	Theory dropping	Feedback is mentioned in abstract, introduction, or methods (with or without citation) but not revisited later
	Theory positioning	Feedback is referred to in the beginning or end (with or without citation) to give meaning to original research results, but the theory did not explicitly inform the design or analysis of the study/educational intervention.
Moderate	Theory mapping	Feedback contributes significantly/explicitly to the design of the study/educational intervention, but not data analysis
Major	Theory application	Employs the concept of feedback throughout, typically to inform study/educational intervention design and analysis
	Theory testing	Empirically validating or testing an existing theory or instrument of feedback
	Theory generation	Building, revising, or expanding a theory to create a new theory

**Table 2:** Summary of degree program and country by curricular setting and theory talk categorization.

	Curricular Setting (Moderate/Major Theory Talk)				
Degree Country	Didactic (24/12)	OSCE/Simulation (5/2)	Experiential (7/5)	Co-Curriculum (9/0)	IPE (2/1)
BPharm	1/2	3/0	0/1	1/0	0/0
Australia	0/1	2/0	-	1/0	-
Japan	1/0	-	-	-	-
Jordan	0/1	1/0	-	-	-
Malaysia	-	-	0/1	-	-
MPharm	4/4	1/0	0/0	1/0	1/0
Australia	-	-	-	1/0	-
Germany	1/2	-	-	-	-
Ireland	1/0	-	-	-	-
United Kingdom	2/2	1/0	-	-	1/0
PharmD	19/6	1/1	4/4	7/0	1/1
Canada	1/0	-	-	-	-
Saudi Arabia	-	-	1/0	-	-
United States	18/6	1/1	3/4	7/0	1/1
Post-graduate	0/0	0/1	1/0	0/0	0/0
Australia *	-	0/1	-	-	-
Ireland *	-	-	1/0	-	-
Multi-level	0/0	0/0	2/0	0/0	0/0
Australia ^†^	-	0/0	1/0	-	-
United States ^‡^	-	0/0	1/0	-	-

* Master of Pharmacy graduates. ^†^ Bachelor of Pharmacy second and final-year students. ^‡^ Doctor of Pharmacy students and graduates. OSCE: objective structured clinical examination; IPE: interprofessional education; BPharm: Bachelor of Pharmacy; MPharm: Master of Pharmacy; PharmD: Doctor of Pharmacy.

**Table 3:** Summary of focused area and method of feedback by curricular setting and theory talk categorization.

	Curricular Setting (Moderate/Major Theory Talk) *			
Focus Area of Feedback	Didactic	OSCE	Experiential	Co-Curricular
Communication	16/7	3/2	3/1	6/0
Patient work up	7/5	1/1	4/1	-
Career/Interview prep	-	-	-	6/0
Lab practical	1/1	-	-	-
Drug/disease info	2/1	-	0/1	-
Self-assessment/reflection	1/0	-	0/2	2/0
CPR	0/2	-	-	-
Calculations	1/1	-	0/1	-
Student portfolio	-	-	0/0	1/0
Method of Feedback				
Written	11/7	-	1/2	5/0
Verbal	4/2	2/0	-	1/0
Multimodal	2/3	2/2	3/2	3/0

* One article could have more than one focused area of feedback. OSCE: objective structured clinical examination; CPR: cardiopulmonary resuscitation.

## Data Availability

The data presented in this study are available in the above Supplementary Materials.

## Supplementary Materials

The following are available online at https://www.mdpi.com/article/10.3390/pharmacy9020091/s1, Table S1: Executed search strategies, Table S2: Description of included studies in didactic curricular setting, Table S3: Description of included studies in objective structured clinical examina-tion/simulation curricular setting, Table S4: Description of included studies in experiential curricular setting, Table S5: Description of included studies in co-curricular setting.
