# Supplementary material for: Feedback for Learning in Pharmacy Education: A Scoping Review
Source: Pharmacy (Basel). 2021 Apr 23;9(2):91. doi: 10.3390/pharmacy9020091 (PMC8167641; doi:10.3390/pharmacy9020091)
Supplement: Supplementary file 1 [file pharmacy-09-00091-s001.zip › pharmacy-1150015-supplementary.pdf]

**Supplementary Table S1.** Executed search strategies.

Total items before duplicates removed: 4,816 Total items after duplicates removed: 2,636

| Search | Query | Items Found |
|---|---|---|
| | **PubMed Search** | |
| #1 | ("Education, Professional"[Mesh] OR "Education, Pharmacy"[Mesh] OR pre-licensure[tiab] OR intern[tiab] OR interns[tiab] OR internship[tiab] OR internships[tiab] OR resident[tiab] OR residents[tiab] OR residency[tiab] OR residencies[tiab] OR fellow[tiab] OR fellows[tiab] OR fellowship[tiab] OR trainee[tiab] OR trainees[tiab] OR student[tiab] OR students[tiab] OR "Students, Pharmacy"[Mesh]) | 706,520 |
| #2 | (Pharmacy[MeSH] OR "Schools, Pharmacy"[Mesh] OR pharmacy[tiab] OR pharmacists[tiab] OR pharmacist[tiab] OR pharmd[tiab] OR pharmacology[tiab] OR pharmacologist[tiab] OR pharmacologists[tiab]) | 181,903 |
| #3 | ("Feedback"[Mesh] OR Feedback[tw] OR "Formative Feedback"[Mesh]) | 164,813 |
| #4 | #1 AND #2 AND #3 | 778 |
| #5 | #4 AND ("2000"[Date - Publication] : "3000"[Date - Publication]) AND English[Language] | 733 |
| | PubMed after duplicates removed: 721 | |
| | **Embase Search** | |
| #1 | ('Education'/exp OR pre-licensure:ti,ab OR intern:ti,ab OR interns:ti,ab OR internship:ti,ab OR internships:ti,ab OR resident:ti,ab OR residents:ti,ab OR residency:ti,ab OR residencies:ti,ab OR fellow:ti,ab OR fellows:ti,ab OR fellowship:ti,ab OR trainee:ti,ab OR trainees:ti,ab OR student:ti,ab OR students:ti,ab OR 'pharmacy student'/exp) | 1,947,351 |
| #2 | ('pharmacy school'/exp OR pharmacy:ti,ab OR pharmacists:ti,ab OR pharmacist:ti,ab OR pharmd:ti,ab OR pharmacology:ti,ab OR pharmacologist:ti,ab OR pharmacologists:ti,ab) | 223,704 |
| #3 | ('feedback system'/exp OR 'constructive feedback'/exp OR feedback:ti,ab,de,tn) | 231,487 |
| #4 | #1 AND #2 AND #3 | 2,460 |
| #5 | #4 AND [english]/lim AND [2000-2021]/py | 2,379 |
| | Embase after duplicates removed: 1,644 | |
| | **Scopus Search** | |
| #1 | TITLE-ABS("pre-licensure") OR TITLE-ABS("intern") OR TITLE-ABS("interns") OR TITLE-ABS("internship") OR TITLE-ABS("internships") OR TITLE-ABS("resident") OR TITLE-ABS("residents") OR TITLE-ABS("residency") OR TITLE-ABS("residencies") OR TITLE-ABS("fellow") OR TITLE-ABS("fellows") OR TITLE-ABS("fellowship") OR TITLE-ABS("trainee") OR TITLE-ABS("trainees") OR TITLE-ABS("student") OR TITLE-ABS("students") | 1,505,881 |
| #2 | TITLE-ABS("pharmacy") OR TITLE-ABS("pharmacists") OR TITLE-ABS("pharmacist") OR TITLE-ABS("pharmd") OR TITLE-ABS("pharmacology") OR TITLE-ABS("pharmacologist") OR TITLE-ABS("pharmacologists") | 231,325 |
| #3 | TITLE-ABS-KEY("Feedback") | 664,733 |
| #4 | #1 AND #2 AND #3 | 973 |
| #5 | #4 AND (LIMIT-TO (LANGUAGE, "English") AND (INCLUDE (PUBYEAR, 2021-2000) | 918 |
| | Scopus after duplicates removed: 98 | |
| | **Web of Science Search** | |
| #1 | TS=(pre-licensure OR intern OR interns OR internship OR internships OR resident OR residents OR residency OR residencies OR fellow OR fellows OR fellowship OR trainee OR trainees OR student OR students) | 1,150,317 |
| #2 | TS=(Pharmacy OR pharmacists OR pharmacist OR pharmd OR pharmacology OR pharmacologist OR pharmacologists) | 159,651 |
| #3 | TS=(Feedback) | 470,888 |
| #4 | #1 AND #2 AND #3 | 828 |

| | | |
|---|---|---|
| #5 | #4 AND PUBLICATION YEARS: (2021-2000) AND LANGUAGES: (ENGLISH) | 786 |
| | Web of Science after duplicates removed: 173 | |

**Supplementary Table S2.** Description of included studies in didactic curricular setting.

| Author(s), Year<br><br>Country | Objectives of study | Number of learners | Method of feed-back | Focused area of feedback | Results of study |
|---|---|---|---|---|---|
| | | | *Moderate Theory Talk* | | |
| (Beal et al., 2020)<br><br>United States | To determine pharmacy students' preferences for and perceptions of in-person and video evaluations | P1: 152<br>P2: 148<br>P3: 144 | Multi-modal | Patient encounter (counseling, interviews)<br><br>Provider communication | 99.3% of students perceived in-person feedback more positively than video feedback except for "nervousness."<br><br>Students who experienced video technology felt significantly less positive toward video evaluations in terms of quality (total mean difference 1.24 vs. 0.83) and amount (total mean difference 1.14 vs 0.77) of written feedback.<br><br>Although students valued the interaction with a larger, more diverse pool of evaluators that was made possible by video evaluations, they did not view video technology as applicable to their future practice. |
| (Bond, Donohoe, Jakeman, Davis, & Morgan, 2017)<br><br>United States | To implement a mock rounds activity designed to introduce students to case presentations and let them practice presentation skills. | P3: 621 | Written | Patient case presentations | Students scored highly in respectfulness and completion time and lowest in logical flow and ability to answer questions. |
| (Brown, Kotlyar, Conway, Seifert, & St Peter, 2007)<br><br>United States | Integrate an internet-based medical chart system into pharmacotherapy course to facilitate evaluation and feedback processes, foster documentation skills, and prepare students for EMR documentation systems | P3: 158 | Written | SOAP notes | Student performance on documentation improved across the semester.<br><br>Students found the EMR easy to use and helpful. |
| (Buring, Brown, Kim, & Heaton, 2011)<br><br>United States | Evaluate the implementation of motivational interviewing in Doctor of Pharmacy curriculum by assessing student knowledge and perceptions and ability to use | P1-3: 75 | Written | Health behavior change counseling<br><br>Motivational interviewing | Student motivational interviewing performance improved each year.<br><br>Repeated, formative assessments and feedback assist student performance. |

| | | | | | |
|---|---|---|---|---|---|
| | motivational interviewing | | | | |
| (Burnworth, 2013)<br><br>United States | To create, implement, and assess an elective course on the pharmacist's role in rare and interesting disorders and cases. | P2/3: 43 | Written | SOAP notes and oral presentations | All students met or exceeded minimum competency levels on assessments.<br><br>Almost all students rated the course favorably, and course became a popular elective. |
| (Gogineni, Aranda, & Garavalia, 2019)<br><br>United States | To apply Gagnes' instructional design model to teaching liver pharmacotherapeutics. | P2: 84 | Multimodal | SOAP notes | Significant student improvement from pre- to post-knowledge assessment and on SOAP performance with Gagnes' instruction model.<br><br>Course evaluations reflected effectiveness of the instructional format. |
| (Hanya, Yonei, Kurono, & Kamei, 2014)<br><br>Japan | Assess the educational effectiveness of a phased, simulation role-playing communication program. | B4: 158 | Written | Student communication during simulation activities | Deeper reflection levels of communication skills were displayed during transcription stage compared to role-playing with feedback or reviewing videos. |
| (Hernick, 2015)<br><br>United States | Develop a series of active learning modules to improve pharmacy students' performance on summative assessments in immunology and infectious disease. | P1: 146 | Written | Immunology and infectious disease information | Student performance on module questions improved with repeated attempts and was predictive of student performance on summative assessments.<br><br>Module use had the most impact on low performing students. |
| (Kalata & Abate, 2013)<br><br>United States | Assess the use of a student portfolio to provide student feedback on self-assessment skills | P1-2:158 | Written | Self-assessment skills | Differences were found between P1 and P2 students for specific questions in the self-assessment assignment.<br><br>Students and faculty mentors felt the portfolio feedback system helped improve self-assessments. |
| (Kawaguchi-Suzuki, Fuentes, Gibbard, Backus, & Marcus, 2018)<br><br>United States | Assess feasibility of mentored, self-directed learning for case presentation on curricular content and new, non-lectured information. | P2: 187 | Verbal | Case presentation performance | Faculty ranked course activities as valuable for student learning.<br><br>Students ranked the group presentation valuable. |
| (Kelsch & Werremeyer, 2011)<br><br>United States | Assess implementation of a requirement for developing a poster on a public health topic. | P3: 71 | Verbal | Public health research poster development | Grading of posters by faculty was high with 17% getting perfect scores.<br><br>Course was well received by students and faculty. |

| | | | | | |
|---|---|---|---|---|---|
| (Lewis & Sewell, 2007)<br><br>United Kingdom | To examine the effectiveness of providing formative feedback for summative computer-aided assessment | M1: 132 | Written | Simulated pharmacology experiment | Students agreed that the feedback was positive and all tests should have formative feedback. |
| (Maher, Hayden, Strawbridge, Gallagher, & Flood, 2020)<br><br>Ireland | To develop a theory-informed video podcast-based method of providing formative feedback and evaluate student perceptions | M1: 53 | Multi-modal | Pharmacy calculations | Most students had positive thoughts about the video podcasts (76%) saying they were useful (75%), helpful for learning (79%).<br><br>Majority of students preferred receiving both video and typed feedback compared to video feedback only. Typed feedback was used for quick verification while video feedback was used for further explanation and clarification. |
| (Melody, Harris, & Grover, 2018)<br><br>United States | Describe the development and evaluation of a MTM elective and assess changes in students' perceived abilities, values, knowledge, and performance conducting MTM services | P3: 24 | Written | Patient encounter (interviews) | Students reported improved abilities in a survey and reported higher agreement on MTM value statement.<br><br>Course grades improved and indicated achievement of aims. |
| (Mpotos, De Wever, Cleymans, et al., 2013)<br><br>Germany | Test efficiency of CPR training with short self-learning and automated assessment/feedback to achieve pre-defined competency and retention after 5 months | "Novice": 404 | Verbal | CPR skills | 99% of participants achieved competence within 4 sessions.<br><br>48% retention rate of correct CPR technique at 5 months. |
| (Persky, Greene, Anksorus, Fuller, & McLaughlin, 2019)<br><br>United States | Development and implementation of a multiday module on assessing and providing feedback on student cognitive and interpersonal skill development and practice readiness | P1: 75 | Verbal | Patient encounter (interviews and counseling) | Capstone pilot provided insights into students' knowledge and skills and gave them feedback on areas to improve. |
| (Planas & Er, 2008)<br><br>United States | Implement a communication skills development system and evaluate its effectiveness in a clinical communications course | P3: 123 | Multi-modal | Patient encounter (interview)<br><br>SOAP notes | Students' patient communication skills improved as evidenced by course assessment scores. |
| (Pittenger et al., 2019) | To examine the feasibility and | P3: 12 | Verbal | Patient work-up: | Quantitative and qualitative analyses demonstrated that |

| | | | | | |
|---|---|---|---|---|---|
| United States | effectiveness of combining whole-task and guided reflection educational design principles with cloud-based learning technologies to simulate the clinical psychiatric APPE in the classroom to begin to close the theory to practice gap | | | psychiatric pharmacotherapy | students progressed in their readiness for APPEs and gained additional psychiatric pharmacy knowledge and evidence-based medicine |
| (Powers & Bright, 2010)<br><br>United States | To describe how webcams and notebook computers were used as an alternative method to VHS recording for recording students during communication exercises in professional practice development laboratory in a PharmD program | P2: 103 | Verbal | Patient encounters | 61.2% of students reported watching the recorded counseling session outside of class.<br><br>The majority (75%) of teaching assistants reported a perceived time savings with the webcams.<br><br>All teaching assistants and faculty perceived a benefit in providing the students with a digital recording for immediate feedback as compared with a 2-week delay in distributing VHS recordings of patient counseling sessions. |
| (Sibbald, 2011)<br><br>Canada | To create, implement, and assess a course based on 3 critical reasoning principles to develop pharmacy students' skills in literature appraisal, content, metacognition, and assessment | P4: 50 | Verbal | Evidence appraisal | Students reported perceptions and self-assessments showing improvement in their learning approaches, sense of responsibility for individual and community learning, skills, and confidence. |
| (Skelley, Wulz, & Thibodeaux, 2018)<br><br>United States | To evaluate introduction of an EMR simulation activity aligned with steps of the PPCP in ambulatory care elective course | P3: 18 | Written | SOAP notes | Students demonstrated significant changes in their knowledge, skills, and confidence in documenting clinical activities using an EMR.<br><br>Showed the benefits of EMR integration into coursework. |
| (Strohfeldt & Grant, 2010)<br><br>United Kingdom | To introduce a new approach to PBL for a renal therapeutics course. | M2: 100 | Written | Care plan on patient case study | Changes in the course structure and grading schemes better reflected the learning progress of students. |
| (Valdez, Shea, Knutsen, & Hoody, 2014) | Implement a student-directed activities and faculty formative feedback | P1: 160 | Written | Patient encounter (counseling) | Students valued receiving personalized formative feedback from faculty and thought |

| | | | | | |
|---|---|---|---|---|---|
| United States | as methods to develop interviewing, assessment, SOAP note writing, patient presentation, and patient counseling skills | | | | personalized formative feedback should be continued. |
| (Villa, Sprunger, Walton, Costello, & Isaacs, 2020)<br><br>United States | To evaluate a clinical documentation rubric for pharmacotherapy PBL courses using IRR among different evaluators | P2: 234 documents<br>P3: 83 documents | Written (rubric) | SOAP note | The mean initial evaluation score was 9.1 (SD 0.9) and the mean second evaluation score was 9.1 (SD 0.9), with no significant difference found between the two. The overall ICC. was 0.7 across multiple graders, indicating good IRR. |
| | | ***Major Theory Talk*** | | | |
| (Barnett, Gallimore, Kopacek, & Porter, 2014)<br><br>United States | To evaluate student and instructor attitudes regarding consistency of SOAP note grading and feedback following changes in SOAP note submission and grading in Pharmacotherapy Laboratory series | P2: 36<br>P3: 54 | Written | SOAP notes | Electronic feedback provided by instructors promoted improvements with future SOAP notes.<br><br>Facilitated positive and constructive instructor feedback.<br><br>For instructors, mean scores perceived inter- and intra-individual grading consistency improved between the two semesters. |
| (Basheti, Qunaibi, AbuRuz, Samara, & Bulatova, 2013)<br><br>Jordan | Evaluate effectiveness of conducting MMRs and home medication reviews on improving undergraduate pharmacy students' pharmaceutical care skills and clinical knowledge | B5: 133 | Written | Findings and recommendations from MMR and home medication review | Pre- post-course scores on same MMR case improved from 33.5 to 62.9.<br><br>Self-assessment questionnaire all significantly improved. |
| (Basheti, Ryan, Woulfe, & Bartimote-Aufflick, 2010)<br><br>Australia | Investigate whether pharmacy students' anonymous peer assessment of a MMR was constructive, consistent with feedback provided by an expert tutor, and enhanced the student's learning experience; determine whether feedback from students was constructive, whether it was consistent with expert feedback, extent to which the use of peer feedback | B5: 182 | Written | Findings and recommendations from medication management review report | Students gave higher marks compared to experts.<br><br>No difference in quality of feedback.<br><br>Majority of students agreed the activity was useful learning experience. |

| | | | | | |
|---|---|---|---|---|---|
| | enhanced students' learning | | | | |
| (Denton, Madden, Roberts, & Rowe, 2008)<br><br>United Kingdom | To ascertain the value of an electronic feedback freeware compared to handwritten feedback comments | First year PSP: 169 | Written | Practical, numeracy<br><br>Report-writing skills | Handwritten feedback performed worse than electronic feedback in "amount of feedback," "identification of errors," and "highlighting of pleasing aspects." |
| (Haack et al., 2017)<br><br>United States | To evaluate electronic vs paper counseling rubric to facilitate timely student feedback and explore differences in student performance, anxiety, and self-perceived preparedness in high stakes practical exam | P3: 201 | Multi-modal (rubric) | Patient encounters (counseling) | No difference in student performance or anxiety.<br><br>Perceived preparedness was higher in students who used electronic rubric during practice - timing of feedback was the mediating process to increase preparedness.<br><br>Electronic rubrics resulted in more timely receipt of feedback. |
| (Medina, Conway, Davis-Maxwell, & Webb, 2013)<br><br>United States | To determine the amount and type of feedback needed to improve pharmacy students' problem-solving skills using team-based learning and a problem-solving rubric | P2: 108<br>P3 written: 121<br>P3 written & verbal: 121 | Multi-modal (rubric) | SOAP notes | Providing pharmacy students with written and verbal explanations may help them improve their problem-solving skills overall. |
| (Mpotos et al., 2011)<br><br>Germany | Compare self-learning using video and voice feedback and traditional instructor to learn CPR skills | "Novice": 120 | Verbal | CPR skills | Self-learning with voice feedback was noninferior to traditional instructor for compression depth; inconclusive for other skills. |
| (Mpotos, De Wever, Calle, et al., 2013)<br><br>Germany | Assess efficacy of self-learning through video training and computer exercises (Voice feedback) for CPR skills | "Novice": 104 | Verbal | CPR skills | After voice feedback a significant improvement was found for all compression and ventilation related outcome measures, except compression rate.<br><br>Differences between gender and skill success resolved with voice feedback. |
| (Nemec & Dintzner, 2016)<br><br>United States | To evaluate recorded audio feedback to written feedback<br><br>To assess student perceptions regarding use of recorded audio feedback<br><br>To determine | P1: 75 | Multi-modal | Drug information | Audio feedback provided significantly more feedback in regard to word count and cognitive process domain.<br><br>No significant differences between audio and written feedback regarding affective processes.<br><br>Significantly more negative |

| | | | | | |
|---|---|---|---|---|---|
| | impact of providing audio vs written feedback on faculty time | | | | emotion-related words found in written feedback compared to audio, but no differences in positive emotions were noted.<br><br>Students felt audio feedback was more personal than written feedback and audio feedback was more useful for indicating organizational and content issues.<br><br>Providing audio feedback took 1.5 min longer, on average, however students received more feedback. |
| (Nutan & Demps, 2014)<br><br>United States | To evaluate the effectiveness of an online approach to carrying out and providing feedback on formative assessments and assess student preferences | P1: 410 over 5 years | Written | Pharmacy calculations | There were significant improvements from C10 to C12 and C13, but no difference C10 and C14 or C11 and C12-14 in course grade.<br><br>Average C11-C14 course grade was 89.49-90.52%.<br><br>Majority of students preferred online approach compared to Scantron for timely return of assignments, thoroughness of feedback, and overall convenience. |
| (Sherman & Johnson, 2019)<br><br>United States | To determine if the patient care and documentation skills of pharmacy students improved in the P3 year using objectively developed SOAP note grading rubric and standardized patient checklist rubric during sequential system of case scenarios | P3: 126 | Written (rubric) | SOAP notes | Scores for the standardized patient evaluations improved for the 2nd and 3rd cases compared to the first ($F=27.48$, $p<0.0001$).<br><br>The SOAP note rubric use had a statistically significant increase in scores over all three cases.<br><br>There was an increase in scores with each successive case scenario for: Treatment goals, monitoring/follow-up, and total score, but no other sections. |
| (Strohfeldt & Khutoryanskaya, 2015)<br><br>United Kingdom | To introduce a new approach to problem-based learning in a medicinal chemistry practical class for pharmacy students | MPharm: 150 | Written | Medicinal chemistry practical | New PBL design appeared to engage students with feedback and integration of knowledge.<br><br>Average examination grade increased significantly. |

P1: first-year Doctor of Pharmacy; P2: second-year Doctor of Pharmacy; P3: third-year Doctor of Pharmacy; EMR: electronic medical record; SOAP: Subjective/Objective/Assessment/Plan; B4: fourth-year Bachelor of Pharmacy; MTM: medication therapy management; CPR:

cardiopulmonary resuscitation; APPE: advanced pharmacy practice experience; VHS: video home system; P4: fourth-year Doctor of Pharmacy; PPCP: Pharmacist Patient Care Process; PBL: problem based learning; M2: second-year undergraduate Master of Pharmacy; IRR: inter-rater reliability; SD: standard deviation; MMR: medication management review; B5: fifth-year Bachelor of Pharmacy; PSP: pharmaceutical science and pharmacy; C10-14: classes of 2010-2014; MPharm: Master of Pharmacy

**Supplementary Table S3.** Description of included studies in objective structured clinical examination/simulation curricular setting.

| Author(s), Year<br><br>Country | Objectives of study | Number of learners | Method of feedback | Focused area of feedback | Results of study |
|---|---|---|---|---|---|
| | | | *Moderate Theory Talk* | | |
| (Bajis, Chaar, Basheti, & Moles, 2019)<br><br>Jordan | Investigate the impact of an in-classroom simulation- and feedback-driven training activity on students' medication reconciliation skills, self-perceived confidence, and overall student satisfaction | B4-5: 144 | Multi-modal | Medication reconciliation skills | Assessment-based competency scores demonstrated significant improvement in student performance during the activity.<br><br>Self-perceived confidence scores significantly improved after the medication reconciliation training intervention.<br><br>Focus group content analysis yielded positive responses such as students valuing receiving feedback on performance and recommendations for future training. |
| (Evans, Alinier, Kostrzewski, Lefteri, & Dhillon, 2011)<br><br>United Kingdom | Describe the development and design of formative and summative OSCE program across a 4-year curriculum | NA | Multi-modal | 20-station OSCE of real-life pharmacy practice scenarios (focus on communication and problem solving) | The school set up a 20-station circuit of five-minute stations that cover a range of practice-based scenarios.<br><br>The process of setting up the first OSCE is described, including developing and validating stations, staff training, room and equipment logistics, and tutor and student feedback. |
| (Gums, Kleppinger, & Urick, 2014)<br><br>United States | Determine the effectiveness of an individualized teaching method in as pharmacy skills laboratory (formative feedback) | P3: 150 | Verbal | Patient encounter (counseling) | Ophthalmic OSCE station scores were higher after the individual formative feedback intervention. |
| (Kubota et al., 2018)<br><br>Japan | Discuss and evaluate the features of this | P2: 242 | Verbal | Patient communication skills training program | The available evidence suggests that simulated patients are suitable for use in communication-skills training. |

| | | | | | |
|---|---|---|---|---|---|
| | communication-skills training program | | | | |
| (O'Reilly, Moles, Boukouvalas, & El-Den, 2019)<br><br>Australia | To use a novel MHFA assessment approach involving simulated role-plays enacted by people with a lived experience of mental illness and explore MHFA participants' and simulated patients' views of participating in simulated role-plays of mental health crises. | B5: 22 | Verbal | Patient encounter (MHFA) | Student appreciated the ability to receive immediate feedback from someone with a lived experience of mental illness to improve their MHFA communication skills. |
| | | | ***Major Theory Talk*** | | |
| (DeLucenay, Conn, & Corigliano, 2017)<br><br>United States | To compare grades of students receiving immediate feedback verses delayed feedback | P3: 153 | Multi-modal | Patient encounter (counselling) | Although more students preferred immediate feedback, overall grades did not differ based on method of feedback. |
| (Tait et al., 2018)<br><br>Australia | Investigate student experiences, satisfaction, and feedback preferences between three scenario simulation modalities (paper-, actor-, and computer-based) | PG2 : 20 | Multi-modal | Patient encounters (medication history and patient triage) | Participants reported similar levels satisfaction across all three modalities. |

B4-5: fourth- and fifth-year Bachelor of Pharmacy; OSCE: objective structured clinical examination; NA: not applicable; P3: third-year Doctor of Pharmacy; P2: second-year Doctor of Pharmacy; MHFA: mental health first aid; PharmD: Doctor of Pharmacy; APN: advanced practice nursing student; PG2: 2 year post-Master of Pharmacy

**Supplementary Table S4.** Description of included studies in experiential curricular setting.

| **Author(s), Year**<br><br>**Country** | **Objectives of study** | **Number of learners** | **Method of feedback** | **Focused area of feedback** | **Results of study** |
|---|---|---|---|---|---|
| | | | ***Moderate Theory Talk*** | | |
| (Bailey, Curington, Brown, | Implement and assess students' perception of and satisfaction with a | P4: 13 | Multi-modal | MI | Improvements were seen in pre to post test in students' knowledge, perceived ability |

| | | | | | |
|---|---|---|---|---|---|
| Hegener, & Espel, 2017)<br><br>United States | formal orientation training program for APPE students focusing on skills related to MI; determine students' ability in using MI compared to published standards | | | | and perceived need to incorporate MI in practice.<br><br>MI ability showed significant improvements MITI scores increased from week 1 to week 4.<br><br>VASE-R score at week 3 was higher than benchmark standard). |
| (Bates et al., 2016)<br><br>United States | To explore use of pharmacy learners as a means to explain pharmacy services in a LLPM; to examine whether an LLPM environment precludes achievement of knowledge-based learning objectives (using a pre and post experience test); to explore learner perception of the experience | P4: 8<br>PGY1: 1<br>PGY2-Oncology: 7 | Multi-modal | Patient work-up | All learners improved scores from pre to post-test (mean +15%).<br>Students mean +18%<br>Resident mean +12.5%<br><br>All learners "passed" the experience.<br><br>Learner feedback on the experience overall was positive. |
| (Flood, Hayden, Bourke, Gallagher, & Maher, 2017)<br><br>Ireland | Evaluate worked example video podcasts as method of providing feedback to pharmacy interns for an online and formative pharmaceutical calculations assessment | Interns: 162 | Multi-modal | Pharmacy calculations | Majority of students found video podcasts were clear, helpful, easy to understand, and useful as method of feedback.<br><br>Learners felt positively about standard written solutions.<br><br>Distinct benefits for each kind of feedback.<br><br>Clear explanations, step-by-step approach and synchronization of audio and visual information was useful of podcasts. |
| (Korayem & Alboghdadly, 2020)<br><br>Saudi Arabia | To evaluate the impact of integrating SBE into APPE on students' learning outcomes, training costs, and satisfaction | P4: 57 | Written (rubric) | Patient work-up | Mean grades in both SBE and hospital-based blocks were mostly above 90% in all learning outcomes. Cognitive skills and affect learning outcomes mean grades were higher in the simulation-based group.<br><br>This newly structured experience provided fourteen direct patient care training seats per rotation and saved around 25,000 Saudi Riyals per rotation.<br><br>Most of the students being satisfied with the simulation scenarios. |

| | | | | | |
|---|---|---|---|---|---|
| (Muzyk et al., 2020)<br><br>United States | To implement and assess the effectiveness of an IPE course designed to provide pharmacy and nursing students with opportunities to improve their understanding of SUDs, assess their attitudes toward patients with SUDs, receive instruction and feed- back on behavior change counseling, and engage in interprofessional education | P4: 13<br><br>Nursing: 14<br><br>Other: 51 | Verbal | Patient encounters (counseling) | Pharmacy and nursing students demonstrated significant improvements in their attitudes toward patients with SUDs and toward IPE collaboration, as measured by the SAAS and SPICE-R2. Approximately 93% of pharmacy and nursing students counseled a patient with a SUD, with 96% of counseled patients reporting their intent to receive follow-up care. |
| (Nisly, Nifong, Coble, & Mihm, 2021)<br><br>United States | To assess pharmacy resident mentorship and feedback skills through participation in a longitudinal pharmacy student and resident seminar series | PGY1: 11<br>PGY2: 6<br>HSPA: 4 | Verbal | Provision of student feedback | Student and resident survey responses were similar and provided favorable feedback on resident mentorship. |
| (Wigmore, Collins, Schneider, Arias, & Moles, 2018)<br><br>Australia | Ascertain how students in an experiential setting compared to experienced staff' respond to a childhood fever scenario using a simulated patient interview including assessment and recommendation to the patient | B2: 65<br>B5: 51 | Multi-modal | Patient encounter (interview)<br><br>Patient assessment, and recommendation | Second year students performed better than staff at questioning.<br><br>Fifth year and staff achieved appropriate outcomes (92 and 65%) with smaller proportion of second year students performing well (52%).<br><br>Staff achieved improvements over time in median questioning scores from feedback provided. |
| | | | ***Major Theory Talk*** | | |
| (Andrus et al., 2018)<br><br>United States | To develop and establish validity for a grading rubric to evaluate diabetes SOAP note writing in primary care APPEs and to assess reliability and student perceptions of the rubric. | P4: 51 | Written (rubric) | SOAP notes | Student scores on SOAP notes increased over the 10 month period (3 SOAP notes per student).<br><br>Correlations exist between scores and APPE grade, pharmaceutical care ability profile scores, and global impression scores.<br><br>For faculty scorers, inter-rater reliability (0.59-fair), intra-rater reliability (0.98-1.00-excellent).<br><br>Students stated rubric improved their ability (84.9%) and |

| | | | | | |
|---|---|---|---|---|---|
| | | | | | confidence (92.4%) on SOAP note writing. |
| (Blackmer, Thompson, Brunner, Patel, & Saseen, 2018)<br><br>United States | To provide a novel culminating experience (2-week intersession course after 5 of 7 APPEs) that assesses student competence and achievement in 5 curricular ability-based outcomes during the P4 year | P4: 149 | Written | Student competence of 4 ability based curricular outcomes using clinical case, drug information, clinical pearl, reflection | 97% of students achieved ability-based outcomes; using the Kirkpatrick model: 93% of students and 100% of faculty felt the course was valuable.<br><br>80% of students and 85% of faculty felt learning occurred from the course.<br><br>Student feedback by faculty led to increased student performance on pre-work compared to in-class work.<br><br>The course complemented learning from previous courses and met the intended purpose. |
| (Chiau, Ali, Bakry, Azmi, & Paraidathatu, 2016)<br><br>Malaysia | Evaluate the effect of video recording feedback in comparison to verbal feedback in communication and counseling among pharmacy students | B4: 45 | Multi-modal | Patient encounter (counseling) | Significant differences in total score, general score, and interpersonal score between first and second counselling sessions for both video and verbal groups were noted but not for control group.<br><br>communication skills in video and verbal group were improved compared to control group.<br><br>Academic performances (cumulative GPA) did not influence the results however gender has significant influence on student performance in general communication (females had higher scores on the second video following feedback). |
| (Nguyen, Wong, Wang, & Goldberg, 2019)<br><br>United States | To evaluate and compare students' ability to perform in each area of the SOAP note during APPEs within an academic year<br><br>To compare the quality of SOAP notes between semesters: summer (1-3 rotation blocks), fall (4-6 rotation blocks), and spring (7-9 rotation blocks) | P4: 128 | Written | SOAP notes | No difference in SOAP note performance based on time of year of APPE.<br><br>Students did better on SOAP note 2 than the first SOAP note after feedback was provided (S/O 3.8 vs 4.2 p=0.0015; A 3.3 vs 3.6 p=0.0072; P 3.6 vs 4 p=0.0007 |

| | | | | | |
|---|---|---|---|---|---|
| (Teply, Spangler, Klug, Tilleman, & Coover, 2016)<br><br>United States | To investigate whether instruction and feedback on reflective responses are beneficial in developing pharmacy students to become more reflective practitioners. | P4: 34 | Written | Self-reflections (instruction on how to do reflections/ intervention group vs no instruction: control group) | Comparing the control to the intervention group, the intervention group had a significantly higher proportion of responses rated as reflective compared to the control group, 83.3% compared to only 37.5% from the control group (p=0.006). Based on the analysis of the pre/post-APPE surveys and the study group assignment, the only factor found to have a significant effect on whether a student's week 4 response was categorized as reflective was the assignment to the intervention group (p=0.011). |

APPE: advanced pharmacy practice experience; MI: motivational interviewing; P4: fourth-year Doctor of Pharmacy; MITI: motivational interviewing treatment integrity; VASE-R: video assessment of simulated encounters-revised; LLPM: layered learning practice model; PGY1: post-graduate year 1; PGY2: post-graduate year 2; IPE: interprofessional education; SUD: substance use disorder; HSPA: health-systems pharmacy administration SAAS: substance abuse attitudinal scale; SPICE-R2: Student Perception of Interprofessional Clinical Education; B2: second-year Bachelor of Pharmacy; B5: fifth-year Bachelor of Pharmacy; SOAP: subjective/objective/assessment/plan; B4: fourth year Bachelor of Pharmacy; GPA: grade point average.

**Supplementary Table S5.** Description of included studies in co-curricular setting.

| **Author(s), Year**<br><br>**Country** | **Objectives of study** | **Number of learners** | **Method of feedback** | **Focused area of feedback** | **Results of study** |
|---|---|---|---|---|---|
| | | | ***Moderate Theory Talk*** | | |
| (Buckley et al., 2020)<br><br>United States | To determine student pharmacists' perceptions of a leadership development program for student organization officers and report the changes in their EIA scores | P1: 166 over 3 years | Written | Student reflections/ emotional intelligence | Each cohort's final mean overall, self-awareness, self-management, and social awareness EIA scores were higher than their corresponding mean initial scores.<br><br>All respondents (61%) either strongly agreed or agreed that participating in the program enhanced their leadership skills. The majority of respondents additionally rated each of the program's activities as being either beneficial or very beneficial. The emotional intelligence assessment and strengths-based leadership assessment were the activities that were most frequently cited as being very beneficial. |
| (Bushell, Austin, Moore, | To determine if formative assessment is an effective method for | B5: 9 | Written | Interview prep | 8/9 students reported the formative assessment task helped them identify their |

| | | | | | |
|---|---|---|---|---|---|
| Hendry, & Adams, 2015)<br><br>Australia | increasing students' odds of obtaining an intern pharmacist position | | | | areas of weakness and allowed them to prepare in their actual interviews.<br><br>8/9 indicated that the feedback helped them perform in their actual interviews. |
| (Caballero et al., 2012)<br><br>United States | To develop a RIPS | P4: 10 | Multi-modal | CV and personal statements<br><br>Journal club<br><br>Verbal case presentation | Students stated the course improved their application materials, interview skills, and confidence in their ability to obtain a residency.<br><br>78% of RIPS participants matched with a residency program. |
| (Knutsen, Hanselin, Lalama, & Moote, 2018)<br><br>United States | Evaluate value of a residency bootcamp in preparing for residency application | P4: 21 | Multi-modal | Post-grad training prep | Students ranked bootcamp experience from 4.62 to 4.69 out of 5 in various areas. |
| (Koenigsfeld et al., 2012)<br><br>United States | Assess student opinion about mock interview activity for residency and impact on match rates | P4: 25 | Verbal | Interview prep | 92% of students felt the mock interview improved their skills.<br><br>88% felt they understood interview questions better.<br><br>Match rates higher in mock interview group compared to national rates. |
| (Lucas, Gibson, & Shum, 2019)<br><br>Australia | To assess pharmacy students' perceptions of the benefits and utility of a novel online reflective-writing tool | M2: 39 | Written | Reflective writing | A comparison of students' responses on the pretest with those on the posttest, which was administered four weeks after the workshop, suggest a noticeable increase in agreement with AWA's benefits as an effective, online tool for improving their reflective learning skills. |
| (Medina, Schwier, Miller, Miller, & Skrepnek, 2018)<br><br>United States | Assess student knowledge of six career skills areas, and perceptions/confidence in these skills | P4: 106 | Multi-modal | CV/resume/interviewing skills<br><br>Business attire/dining etiquette<br><br>Networking | Knowledge improved by 7.1 points over baseline.<br><br>Confidence improved in career skills areas. |
| (Ray, DuBrava, & Jacks, 2020)<br><br>United States | To create a portfolio meeting multiple needs, including learning improvement, curricular | P1-4: 103 | Written (rubric) | Student portfolio | This course provides a wealth of opportunity for student growth and generation of assessment data, but it is best suited for programs with |

| | | | | | |
|---|---|---|---|---|---|
| | assessment, and accreditation | | | | ample support resources to maintain its integrity as designed. |
| (Wolfgang, 2019)<br><br>United States | Describe a comprehensive design of mock residency interviews intended to closely mimic the environment and variety of interview settings commonly seen in post-graduate year 1 interviews | P4: 24 | Written | Interview prep | Precise scheduling and widespread participation of faculty and pharmacists is key for success of interview prep program. |

EIA: Emotional Intelligence Appraisal; P1: first-year Doctor of Pharmacy; B5: fifth-year Bachelor of Pharmacy; RIPS: residency interviewing preparatory seminar; P4: fourth-year Doctor of Pharmacy; CV: curriculum vitae; M2: second/final-year Master of Pharmacy; AWA: Academic Writing Analytics.
